# Electromagnetic Duality and Exceptional-Point Transitions in Non-Hermitian RH/LH LC Bilayers

**DOI:** 10.3390/ma19163513

**Published:** 2026-08-19

**Authors:** L. Palma-Chilla, Juan A. Lazzús, J. C. Flores

**Affiliations:** 1 Departamento de Física, Universidad de La Serena, Casilla 554, La Serena 1700000, Chile; 2 Departamento de Física, FACI, Universidad de Tarapacá, Casilla 7-D, Arica 1000000, Chile

**Keywords:** non-Hermitian metamaterials, electromagnetic duality, exceptional points, LC networks, mode conversion

## Abstract

An analytical non-Hermitian bilayer composed of coupled right-handed and left-handed LC lattices is investigated. The uncoupled lattices exhibit exact dual dispersions characterized by complementary direct and inverse excitation spectra. When the layers are coupled through a purely imaginary interlayer coupling, the resulting non-Hermitian Hamiltonian undergoes exceptional-point transitions that separate regions with purely real eigenenergies from regions with complex eigenenergies. It is shown that the spectral splitting is progressively transferred from the real-energy sector to the imaginary-energy sector, giving rise to hybridized modes with finite lifetimes. The corresponding relaxation time, which is directly related to the mobility of the excitations, is governed by the imaginary-energy splitting and diverges at the exceptional-point boundary. These findings establish a direct connection between electromagnetic duality, non-Hermitian spectral properties, and transport phenomena in mesoscopic networks. Possible extensions of the present approach may be relevant for artificial circuit platforms, including Josephson-junction arrays.

## 1. Introduction

Non-Hermitian systems have attracted considerable attention due to their ability to describe open physical systems with energy gain/loss and finite lifetimes [1,2]. In parallel, LC metamaterials provide versatile platforms for realizing synthetic dispersions and engineered transport properties through purely electrical architectures [3,4]. In particular, right-handed (RH) and left-handed (LH) metamaterials exhibit fundamentally different propagation characteristics, such as positive- and negative-index responses arising from their complementary electromagnetic behavior [5,6,7].

The combination of RH and LH structures into coupled bilayer architectures opens the possibility of generating dual spectra, exceptional points, non-Hermitian transitions, and unconventional transport phenomena [1,2,3,4,5,6,7,8,9,10,11,12,13,14,15]. Such systems also provide a natural framework for investigating the interplay between electromagnetic duality and non-Hermitian spectral hybridization. In coupled systems, it is useful to distinguish between coherent and dissipative mode coupling. While a conventional coherent interaction is generally represented by a real off-diagonal coupling and primarily produces a splitting of the real frequencies or energies, dissipative or imaginary coupling can redistribute the spectral splitting between the real and imaginary parts of the eigenvalues and give rise to exceptional-point transitions [16,17]. Related effects have been extensively investigated in non-Hermitian resonators and passive PT-symmetric coupled systems [1].

**Figure 1:**
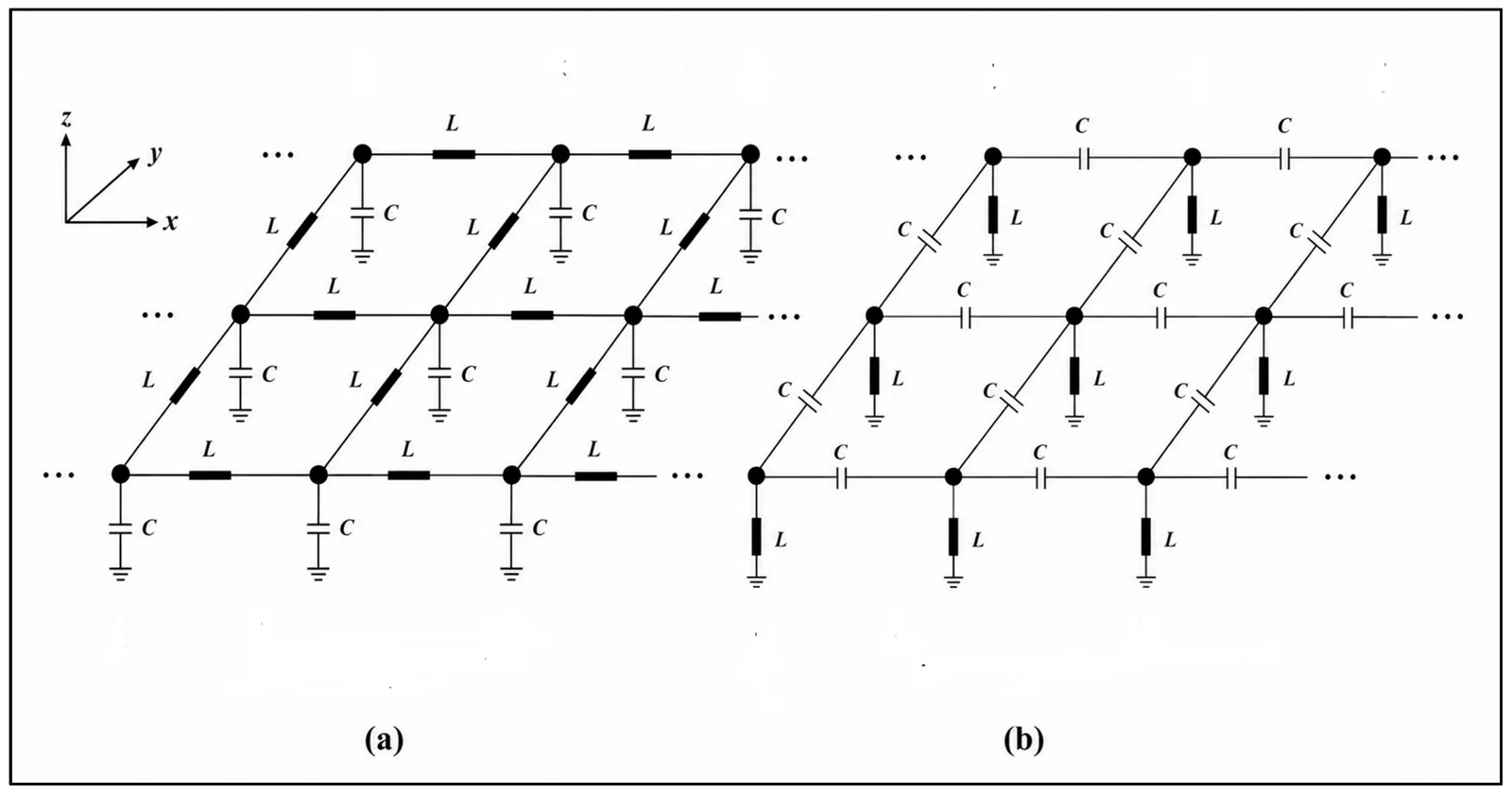
Schematic representation of the isolated metamaterial plates forming the RH/LH bilayer. (**a**) Right-handed (RH) plate composed of inductors L connected in series along the x and y directions, with grounding capacitors C connected from each lattice node to the ground plane. The lattice spacing is a. (**b**) Left-handed (LH) dual plate composed of capacitors C connected in series along the lattice directions, with grounding inductors L connected from each lattice node to the ground plane. The same lattice spacing a is used in both plates.

In this work we introduce a bilayer system formed by the coupling between a RH LC plate and a LH LC plate (see Figure 1). Starting from a discrete LC network equation derived from Kirchhoff’s laws, we obtain exact analytical expressions for the isolated RH and LH spectra. We then construct a non-Hermitian bilayer Hamiltonian and derive its exact eigenvalues, together with relaxation-time effects and their implications for excitation mobility. The distinctive feature of the present system is that the two coupled components are not arbitrary resonators: they are electromagnetically dual RH and LH lattices, whose spectra are related by the exact transformation L↔C. Consequently, the non-Hermitian coupling acts between two spectrally constrained and complementary branches rather than between two independent modes.

Mesoscopic superconducting networks may admit an effective quantum description in terms of collective modes. Within such a framework, the modal frequencies can be mapped onto an effective spectral energy through the correspondence E=ℏω, allowing the use of band-structure methods and non-Hermitian spectral analysis [13]. This perspective suggests possible connections with engineered platforms such as superconducting Josephson arrays and circuit-QED metamaterials, where quantized collective excitations emerge from coupled LC architectures [14,15].

The present RH/LH bilayer therefore constitutes a mesoscopic metamaterial whose spectral properties can be derived analytically. Although exceptional points are not exclusive to RH/LH systems, the RH/LH duality determines the separation and complementary structure of the uncoupled branches and, consequently, the location of the exceptional-point boundaries. In particular, the dual dispersions lead to a characteristic finite interval in which the eigenvalues become complex, defining a duality-controlled real-to-complex spectral transition. This provides a specific physical consequence of the RH/LH architecture beyond the generic behavior of a non-Hermitian two-mode system.

## 2. Isolated Right-Handed and Left-Handed Plates

### 2.1. Right-Handed Plate

We first consider an infinite two-dimensional right-handed (RH) LC plate composed of identical inductors L connected in series along the x and y directions, while each lattice node is connected to ground through an identical capacitor C (see Figure 1a). The lattice spacing is denoted by a.

For periodic LC networks, the normal-mode spectrum can be obtained from the impedance formulation of the discrete lattice. The corresponding dispersion relation is given by [10]

(1) $$\sum_{j}\frac{\sin^{2}\left(k_{j}a/2\right)}{Z_{j}}=0,$$

where j=x,y,z labels the three lattice directions. Here, $Z_{x}$ and $Z_{y}$ are the impedances associated with the inter-site couplings in the xy-plane, while $Z_{z}$ represents the grounding impedance connected to each node. The wave numbers $k_{x}$ and $k_{y}$ describe Bloch propagation along the lattice plane, whereas the z-direction is treated as a single fundamental mode.

For the RH plate,

(2) $$\left\{\begin{array}{c} Z_{x}=Z_{y}=i\omega L,\\ Z_{z}=\frac{1}{i\omega C}. \end{array}\right.$$

Substituting these impedances into Equation (1) leads to the exact RH dispersion relation

(3) $$\omega \left(k_{x},k_{y}\right)=\frac{2}{\sqrt{LC}}\sqrt{\sin^{2}\left(\frac{k_{x}a}{2}\right)+\sin^{2}\left(\frac{k_{y}a}{2}\right)}.$$

Using the effective quantum correspondence E=ℏω and the natural spectral scale $E_{0}=\frac{\hbar }{\sqrt{LC}}$, the RH spectrum can be written as

(4) $$E_{RH}\left(k_{x},k_{y}\right)=2E_{0}\sqrt{S\left(k_{x},k_{y}\right)},$$

where S is a dimensionless lattice structure function given by

(5) $${S\left(k_{x},k_{y}\right)=\sin}^{2}\left(\frac{k_{x}a}{2}\right)+\sin^{2}\left(\frac{k_{y}a}{2}\right).$$

Within the first Brillouin zone ($-\pi /a\leq k_{x},k_{y}\leq \pi /a$), the structure function ranges from 0≤S≤2, valid for wave-lengths λ≥2a, and this definition is applied throughout the present analysis.

### 2.2. Left-Handed Plate

We now consider the dual left-handed (LH) plate, consisting of identical capacitors C connected in series along the x and y directions, while each lattice node is connected to ground through an identical inductor L. This structure constitutes the exact electromagnetic dual of the RH network discussed previously (see Figure 1b).

Thus, the LH plate is obtained by interchanging the inductive and capacitive impedances

(6) $$\left\{\begin{array}{c} Z_{x}=Z_{y}=\frac{1}{i\omega C},\\ Z_{z}=i\omega L. \end{array}\right.$$

Following the same procedure used for the RH plate via Equation (1), the exact LH dispersion relation becomes

(7) $$\omega \left(k_{x},k_{y}\right)=\frac{1}{2\sqrt{LC}}\frac{1}{\sqrt{\sin^{2}\left(\frac{k_{x}a}{2}\right)+\sin^{2}\left(\frac{k_{y}a}{2}\right)}}.$$

Using the effective correspondence E=ℏω, the spectral scale $E_{0}=\frac{\hbar }{\sqrt{LC}}$, and the auxiliar variable $S\left(k_{x},k_{y}\right)$ the LH spectrum becomes

(8) $$E_{LH}\left(k_{x},k_{y}\right)=\frac{E_{0}}{2}\frac{1}{\sqrt{S\left(k_{x},k_{y}\right)}}.$$

**Figure 2:**
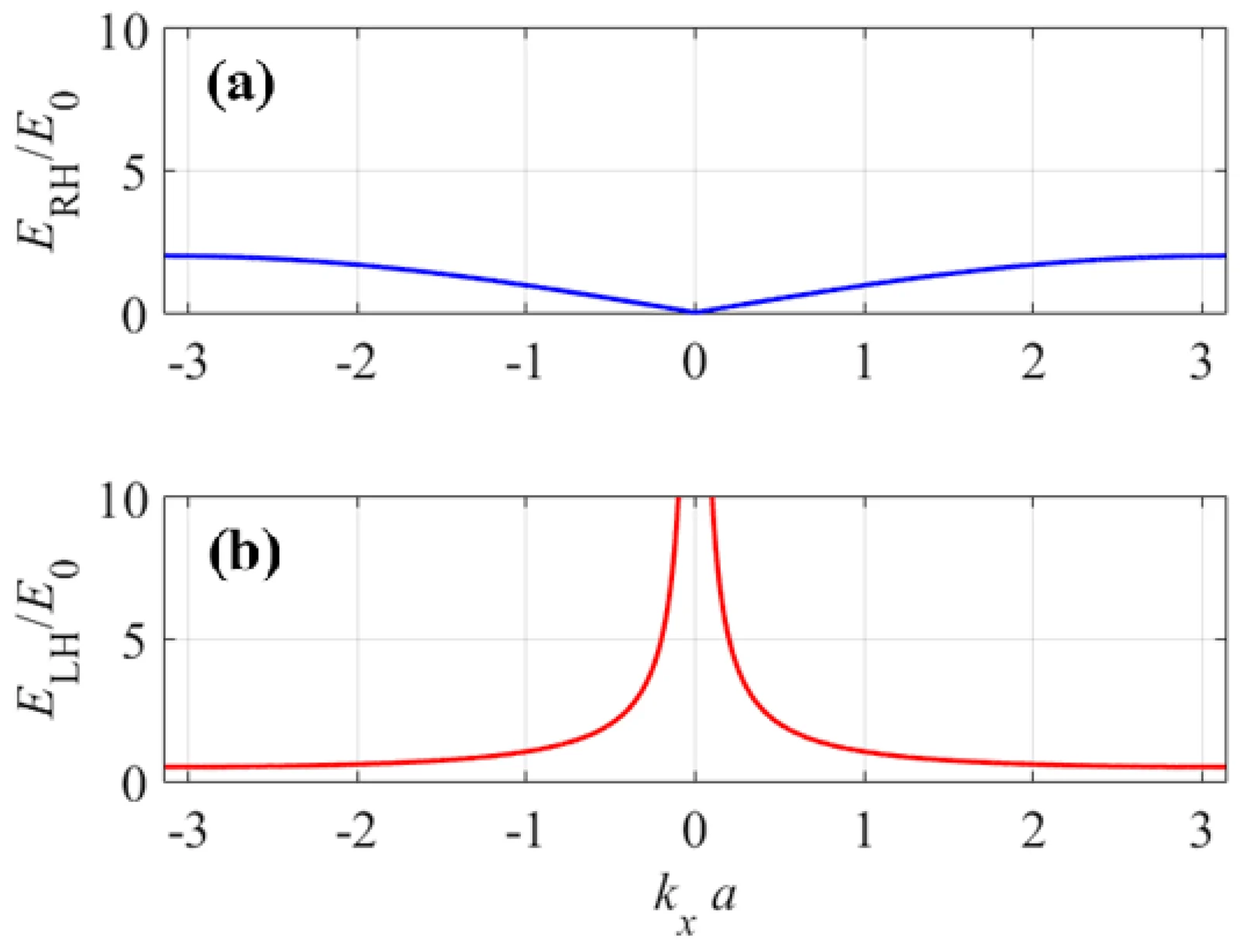
Dimensionless non-coupled isolated spectra $E_{RH}/E_{0}$ and $E_{LH}/E_{0}$ as functions of $k_{x}a$, evaluated along the one-dimensional cut $k_{y}a=0$ of the first Brillouin zone. (**a**) The RH spectrum forms a bounded periodic band with minima at $k_{x}a=0$ and maximum energy near the zone boundaries. (**b**) The LH spectrum exhibits the complementary inverse behavior, presenting a strong enhancement at $k_{x}a\sim 0$ and progressively decreasing toward the zone boundaries.

Figure 2 shows the exact isolated spectra of the RH and LH plates obtained from the full lattice dispersions. The RH branch exhibits a bounded periodic spectrum characteristic of direct LC propagation, whereas the LH branch displays the corresponding dual inverse behavior.

### 2.3. RH/LH Duality

The isolated spectra satisfy the exact duality relation [12]

(9) $$E_{RH}E_{LH}=E_{0}^{2}.$$

Equivalently,

(10) $$2\sqrt{S\left(k_{x},k_{y}\right)}⟷\frac{1}{2\sqrt{S\left(k_{x},k_{y}\right)}}.$$

This dual transformation maps high-frequency RH modes into low-frequency LH modes while preserving the characteristic spectral scale $E_{0}$. The resulting RH/LH bilayer will therefore be interpreted a coupled structure composed of mutually dual spectral branches.

This RH/LH duality constitutes the fundamental symmetry underlying our bilayer system. In Figure 2, both spectra satisfy the duality relation (Equation (10)), revealing the complementary nature of the direct and dual metamaterial plates across k.

## 3. Non-Hermitian RH/LH Bilayer

For a fixed k, the isolated RH and LH plates each contribute a single collective mode characterized by the dispersions $E_{RH}$ and $E_{LH}$. The bilayer dynamics can therefore, be projected onto the reduced modal basis $\left\{\left|\left.RH\right\rangle ,\right.\left.\left|LH\right.\right\rangle \right\}$, leading to an effective two-level description analogous to coupled-mode formulations commonly used in transmission-line and mesoscopic resonator systems. Thus, this interlayer interaction hybridizes the RH and LH branches.

In the present model, the RH/LH conversion is assumed to be mediated by an effective non-Hermitian interlayer coupling implementing the electromagnetic duality transformation L↔C [18,19]. The resulting Hamiltonian is an effective two-mode description of an open bilayer system, in which the non-Hermiticity is introduced through the off-diagonal coupling between the RH and LH plates. Physically, the imaginary coupling can be interpreted as an effective interaction mediated by external dissipative degrees of freedom, or a reservoir, whose dynamics are not explicitly included in the model for simplicity. Within this effective description, the influence of these external degrees of freedom is incorporated phenomenologically through the assumed non-Hermitian interlayer coupling. This effective coupling provides a mechanism for exchanging collective excitations between the two plates while introducing finite-lifetime effects associated with the open character of the system. The resulting non-Hermitian interaction is characterized by the coupling energy G, with the imaginary off-diagonal terms representing the effective dissipative RH/LH mode conversion and constituting the origin of the non-Hermitian character of the bilayer. Introducing the dimensionless coupling parameter $g=G/E_{0}$, the bilayer Hamiltonian can be written as

(11) $$H\left(k_{x},k_{y}\right)=E_{0}\left(\begin{array}{cc} 2\sqrt{S} & ig\\ ig & \frac{1}{2\sqrt{S}} \end{array}\right).$$

The eigenvalue spectrum follows from the secular equation det(H−EI)=0, yielding

(12) $$E_{\pm }=\frac{E_{0}}{2}\left[\left(2\sqrt{S}+\frac{1}{2\sqrt{S}}\right)\pm \sqrt{{\left(2\sqrt{S}-\frac{1}{2\sqrt{S}}\right)}^{2}-4g^{2}}\right].$$

**Figure 3:**
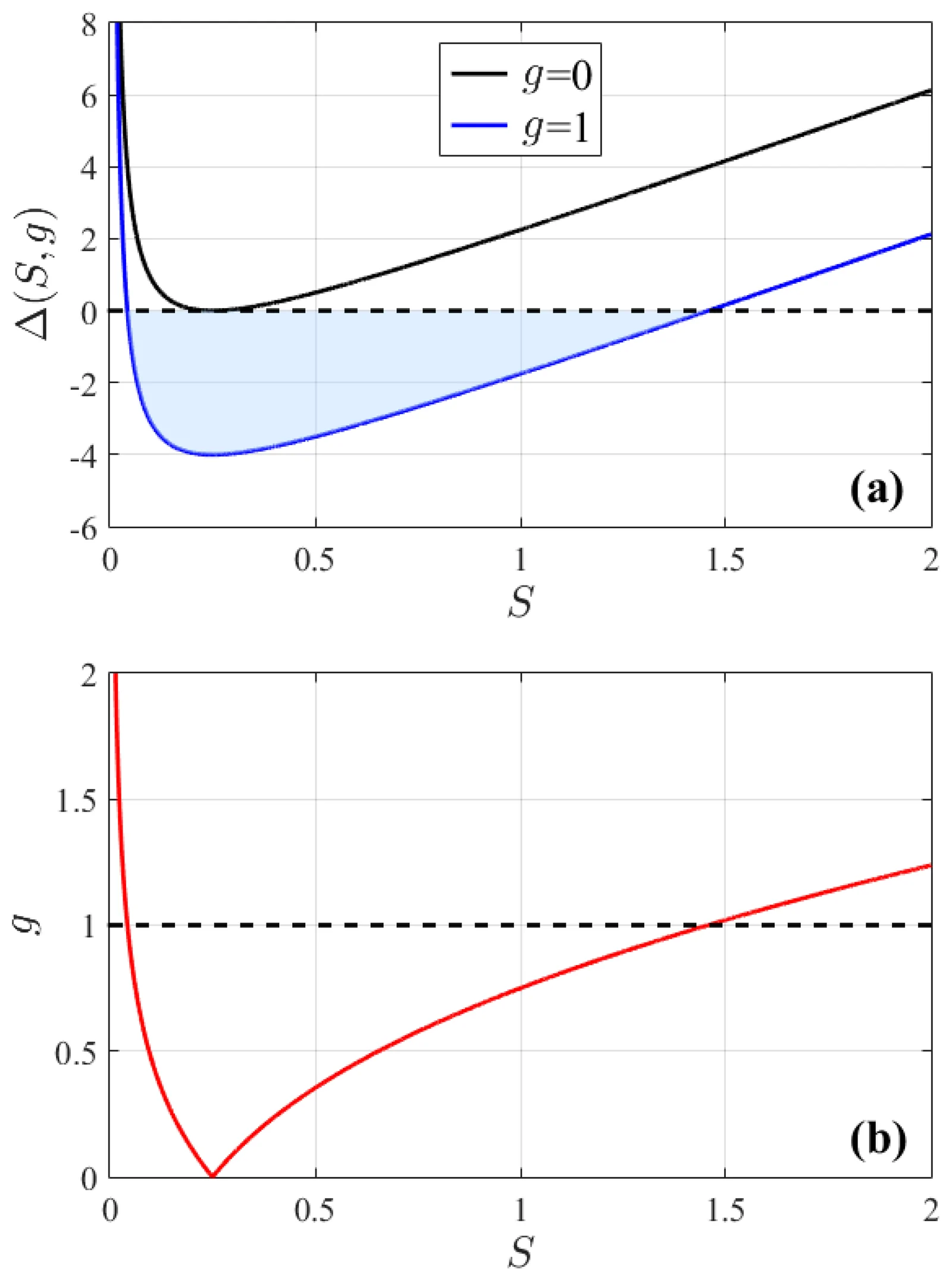
(**a**) The discriminant Δ determines the crossover between real and complex energy eigenvalues of the proposed bilayer LC system (Equation (12)). The case g=1 is shown as an illustrative example and compared with the uncoupled case g=0. The blue shaded area indicates the finite interval where Δ<0, corresponding to complex energy eigenvalues. Within this regime, the relaxation time is finite and is determined by the imaginary part of the eigenvalues. For any value of g, the discriminant reaches its minimum value at S=1/4 where $\Delta_{min}=-4g^{2}$. In this case ($g=1<7\sqrt{2}/8\sim 1.24$) the exceptional points, where Δ=0, are $S_{-}\sim 0.05$ and $S_{+}\sim 1.46$. (**b**) Exceptional-point diagram obtained from the condition Δ=0. The curve g(S) separates the purely real (Δ>0) and complex (Δ<0) spectral regimes. For a given coupling g, the intersections at $S_{-}$ and $S_{+}$ define the boundaries of the complex energy region. The horizontal dashed line corresponds to g=1. In both panels, The physically allowed range of the lattice structure function is 0≤S≤2.

Defining the discriminant

(13) $$\Delta \left(S,g\right)={\left(2\sqrt{S}-\frac{1}{2\sqrt{S}}\right)}^{2}-4g^{2},$$

the spectral properties of the bilayer are entirely determined by its sign. Here, S is defined by Equation (5). The exceptional-point condition is therefore constrained by the physically allowed domain of the lattice structure function. Figure 3 summarizes the behavior of the discriminant and the corresponding exceptional-point boundaries. Figure 3a shows the discriminant Δ as a function of the variable S, identifying the boundaries between the real- and complex-energy regimes. Figure 3b displays the exceptional-point curve g(S), obtained from the condition Δ=0. For a fixed value of g, two solutions of S (denoted by $S_{\pm }$) are obtained, which delimit the interval where Δ < 0 and the eigenenergies become complex conjugates. As an example, the case g=1 is indicated in Figure 3b, identifying the corresponding complex eigenvalues region $S_{-}<S<S_{+}$.

For Δ>0, the eigenvalues remain purely real and describe stable hybridized collective modes (Figure 3).

For Δ<0, the spectrum becomes complex, $Im\left(E_{\pm }\right)=\frac{E_{0}}{2}\sqrt{-\Delta }$. It is worth noting that, for a given value of g, this condition is fulfilled only over a finite range of S, excluding the singular point S=0 (see Figure 3).

The condition Δ=0 defines the exceptional-points boundary separating the real and complex-energy regimes (called $S_{\pm }={\left(\sqrt{1+g^{2}}\pm g\right)}^{2}/4$). Across this transition, the eigenvalue splitting is transferred from the real-energy sector to the imaginary-energy sector, producing hybridized modes with finite relaxation rates.

Three important limits must be considered in order to explain some physical results.

(a) In the long-wavelength limit (ka≪1), from Equation (5), one has S~0, and the energy square-root term Equation (12) remains always real. Consequently, the spectrum is purely real in this regime, and no relaxation time exists.
(b) In the vicinity of S~1/4, the square-root term in Equation (12) becomes imaginary. As a result, the spectrum acquires an imaginary component, leading to the emergence of a finite and quantifiable relaxation time (see Section 4).
(c) At the self-dual point S=1/4, for instance when $ak_{x}=\pm \pi /3$ and $ak_{y}=0$, the discriminant reaches its minimum value $\Delta =-4g^{2}$, and Equation (12) reduces to $E_{\pm }=E_{0}\pm ig.$ In this configuration the RH and LH branches become spectrally equivalent, and the imaginary splitting attains its maximum value.

**Figure 4:**
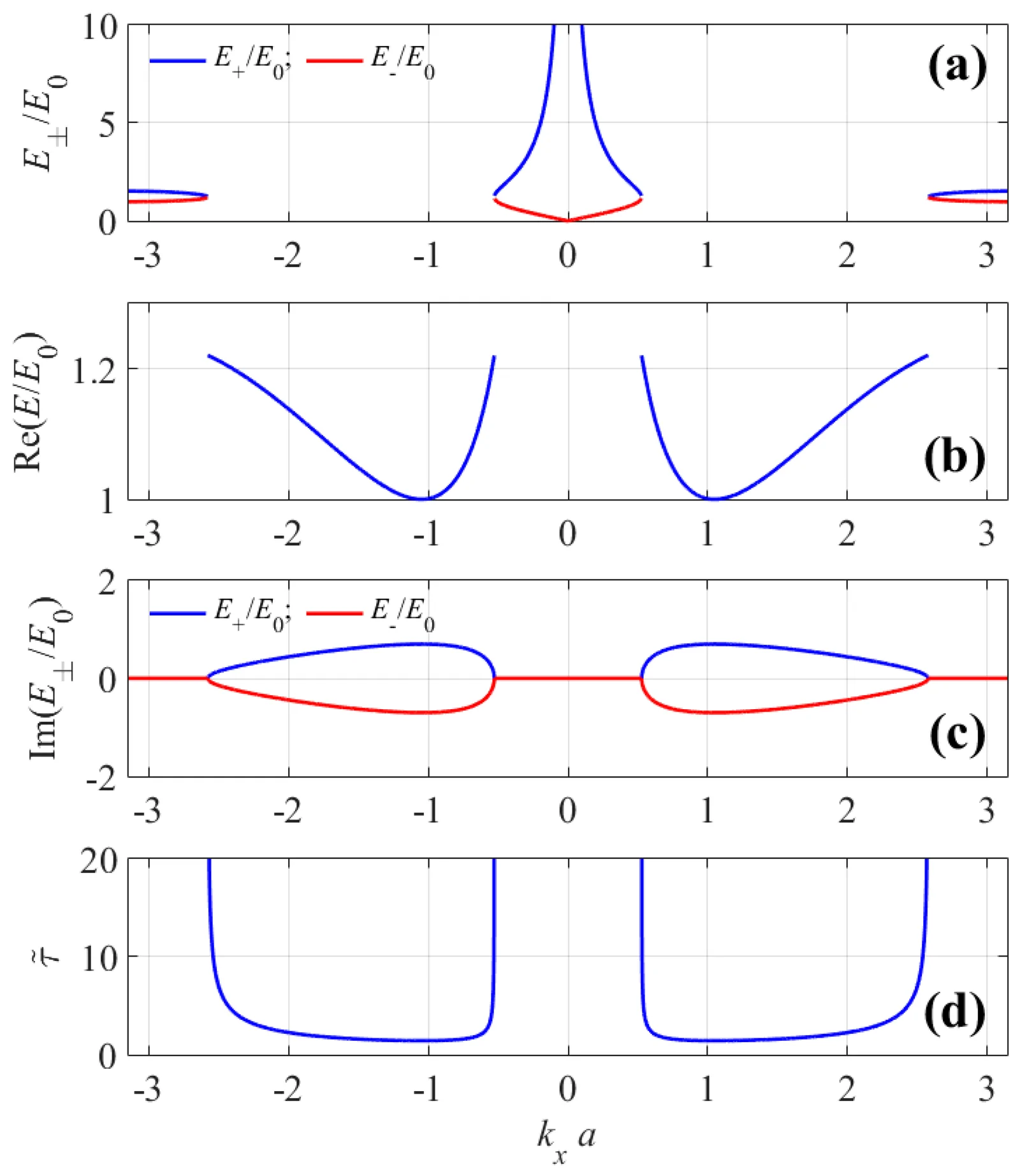
Dimensionless non-Hermitian bilayer spectrum $E_{\pm }/E_{0}$ as a function of the dimensionless wavenumber $k_{x}a$ and $k_{y}a=0$. All panels correspond to the dimensionless non-Hermitian Hamiltonian Equation (11) with coupling parameter $g=G/E_{0}=0.7$. (**a**) Energy branches $E_{\pm }/E_{0}$ for Δ≥0. (**b**) Real-energy branches $Re\left(E/E_{0}\right)$ for Δ<0, showing the continuation of the spectrum through the complex regime. (**c**) Imaginary-energy branches $Im\left(E_{\pm }/E_{0}\right)$ emerging for Δ<0, indicating the onset of the non-Hermitian spectral regime. The branch with $Im\left(E_{+}/E_{0}\right)>0$ (blue) corresponds to the amplifying (growing) mode, whereas the branch with $Im\left(E_{-}/E_{0}\right)<0$ (red) corresponds to the decaying mode, under the convention $\Psi \left(t\right)\propto e^{-iEt/\hbar }$. (**d**) Dimensionless relaxation time $\tilde{\tau }=\;\tau /\tau_{0}$ (with $\tau_{0}\;=\sqrt{LC}$), obtained from the inverse imaginary-energy scale and diverging at the exceptional-point boundaries Δ=0.

Figure 4 shows the exact non-Hermitian bilayer spectrum obtained from Equation (12) for g=0.7. Figure 4a displays the energy branches corresponding to the region Δ>0, where the eigenvalues remain purely real. The apparent gap in the real spectrum around the center of the Brillouin zone does not correspond to a conventional energy band gap, but to an interval in k-space where no purely real eigenvalues exist, since Δ<0. Figure 4b,c show the real- and imaginary-energy branches that emerge for Δ<0, revealing that the real eigenvalue branches continuously coalesce at the exceptional-point boundaries and evolve into complex eigenvalues. Thus, the transition transfers the eigenvalue splitting from the real-energy sector to the imaginary-energy sector, rather than eliminating the corresponding states.

## 4. Relaxation Time

Inside the non-Hermitian regime (Δ<0), the imaginary part of the eigenvalues take the form $\frac{E_{0}}{2}\sqrt{-\Delta }$. Thus, the corresponding relaxation time is

(14) $$\tau =\frac{\hbar }{Im\left(E_{\pm }\right)}=\frac{2\hbar }{E_{0}\sqrt{-\Delta }}.$$

The relaxation time obtained from the imaginary part of the eigenenergy characterizes the electronic excitations in this non-Hermitian regime, which is connected directly with the mobility μ∝τ. Using a conventional transport picture for carriers with effective mass m, this characteristic time may also be related to the mobility through a Drude-like relation μ~eτ/m, providing a qualitative indication of carrier transport. By introducing the expected temporal scale $\tau_{0}\;=\sqrt{LC}$, the relaxation time can be written in dimensionless form as $\tilde{\tau }=\;\tau /\tau_{0}$. Figure 4d shows the dimensionless relaxation time as function of the wavenumber. The relaxation time diverges at the exceptional-point boundary (Δ=0), where the imaginary component of the spectrum vanishes. Consequently, the characteristic decay time of the hybridized RH/LH modes increases strongly as the system approaches the transition between the real and complex-energy regimes. The relaxation time is defined only within the region Δ<0, where the eigenvalues acquire a finite imaginary component. Outside this region (Δ≥0), the spectrum remains purely real and the hybridized modes do not exhibit exponential decay, so no finite relaxation time can be assigned (see Figure 4d).

## 5. Physical Platforms and Experimental Relevance

The proposed RH/LH bilayer may be viewed as a mesoscopic lattice in which coherent and dissipative dynamics coexist through collective excitation processes. This perspective is consistent with recent studies of non-Hermitian coupled-mode systems, where tunable strong coupling can drive the evolution from energy-level anticrossing to linewidth bifurcation and exceptional-point behavior [20,21]. In particular, plasmon–exciton systems have demonstrated experimentally accessible transitions between these regimes, providing a useful physical reference for the real-to-complex spectral transition considered here [20]. Related tunable borophene–perovskite structures have also been shown to exhibit strong coupling and passive PT-symmetry breaking, with the energy splitting disappearing as the coupling conditions are varied [21].

These results provide a useful comparison with the present RH/LH bilayer, while highlighting its distinctive feature: here, the two coupled modes are constrained by an exact electromagnetic duality rather than representing generic resonances. Consequently, the location and structure of the exceptional-point boundaries are determined by the dual RH/LH dispersions and the non-Hermitian interlayer coupling.

Artificial circuit platforms, including Josephson-junction arrays and circuit-QED architectures, offer potential settings in which coupled LC networks with finite dissipation and tunable non-Hermitian couplings can be engineered [14,15]. For characteristic lattice scales in the range $a∼10^{-6}–10^{-3}\;m$, experimentally accessible values of inductance and capacitance typically lead to collective-mode frequencies of the order of $10^{11}–10^{14}\;Hz$, spanning the terahertz regime [22,23,24]. In these systems, the characteristic frequency scale is determined by the circuit parameters through ($\omega_{0}=1/\sqrt{LC}$), so that a given frequency can be realized through different combinations of inductance and capacitance. Josephson-junction transmission lines have been experimentally demonstrated as tunable artificial crystals with controllable microwave band structures [22], while more recent Josephson metamaterials have demonstrated tunable operation in the GHz regime, with amplification bands extending from 4 to 12 GHz [23]. Moreover, Josephson-junction devices have also been developed for sub-terahertz operation [24], providing experimentally accessible frequency scales beyond the conventional microwave regime. Within such a framework, the correspondence $E_{0}=\hbar \omega_{0}=\hbar /\sqrt{LC}$ allows the collective normal modes of the RH/LH bilayer to be interpreted as effective excitations [13]. Consequently, the present model does not require a unique choice of L and C; instead, these parameters can be selected to set the desired characteristic frequency while preserving the dimensionless structure of the RH/LH bilayer. The effective interlayer coupling can then be tuned independently through the corresponding circuit coupling element. This provides a concrete experimental route for implementing the dissipative coupling considered here and for probing the predicted real-to-complex spectral transition and the associated exceptional-point boundaries.

## 6. Conclusions

Our results demonstrate that the RH/LH non-Hermitian bilayer model, Equation (12), behaves as a dual-mode conversion metamaterial. Unlike isolated RH and LH plates (Equations (4) and (8)), which act as complementary dispersive filters, the coupled bilayer enables controlled RH↔LH mode hybridization. Physically, the non-Hermitian interlayer coupling characterized by the dimensionless parameter g (Equation (11)) mixes inductive and capacitive transport pathways, so that the normal modes are no longer confined to a single lattice but become hybridized states distributed across both plates. As a consequence, transport is governed not only by propagation within each network but also by the rate at which energy is exchanged between the dual channels. This fact gives rise to exceptional-point transitions, finite modal lifetimes (Equation (14), related to mobility), and transport channels whose propagation properties can be engineered through the non-Hermitian coupling.

## Data Availability

The original contributions presented in this study are included in the article. Further inquiries can be directed to the corresponding author.
